# Supplementary material for: Ammonia Production by Streptomyces Symbionts of Acromyrmex Leaf-Cutting Ants Strongly Inhibits the Fungal Pathogen Escovopsis
Source: Microorganisms. 2021 Jul 29;9(8):1622. doi: 10.3390/microorganisms9081622 (PMC8400888; doi:10.3390/microorganisms9081622)
Supplement: Supplementary file 1 [file microorganisms-09-01622-s001.zip › microorganisms-1307169-supplementary.pdf]

Supporting Information

Ammonia Production by *Streptomyces* Symbionts of *Acromyrmex* Leaf-cutting Ants Strongly Inhibits the Fungal Pathogen *Escovopsis*

Basanta Dhodary[1] and Dieter Spiteller[1]*

[1]Chemical Ecology/Biological Chemistry, University of Konstanz, Universitätsstrasse 10, 78457 Konstanz, Germany.

*corresponding author

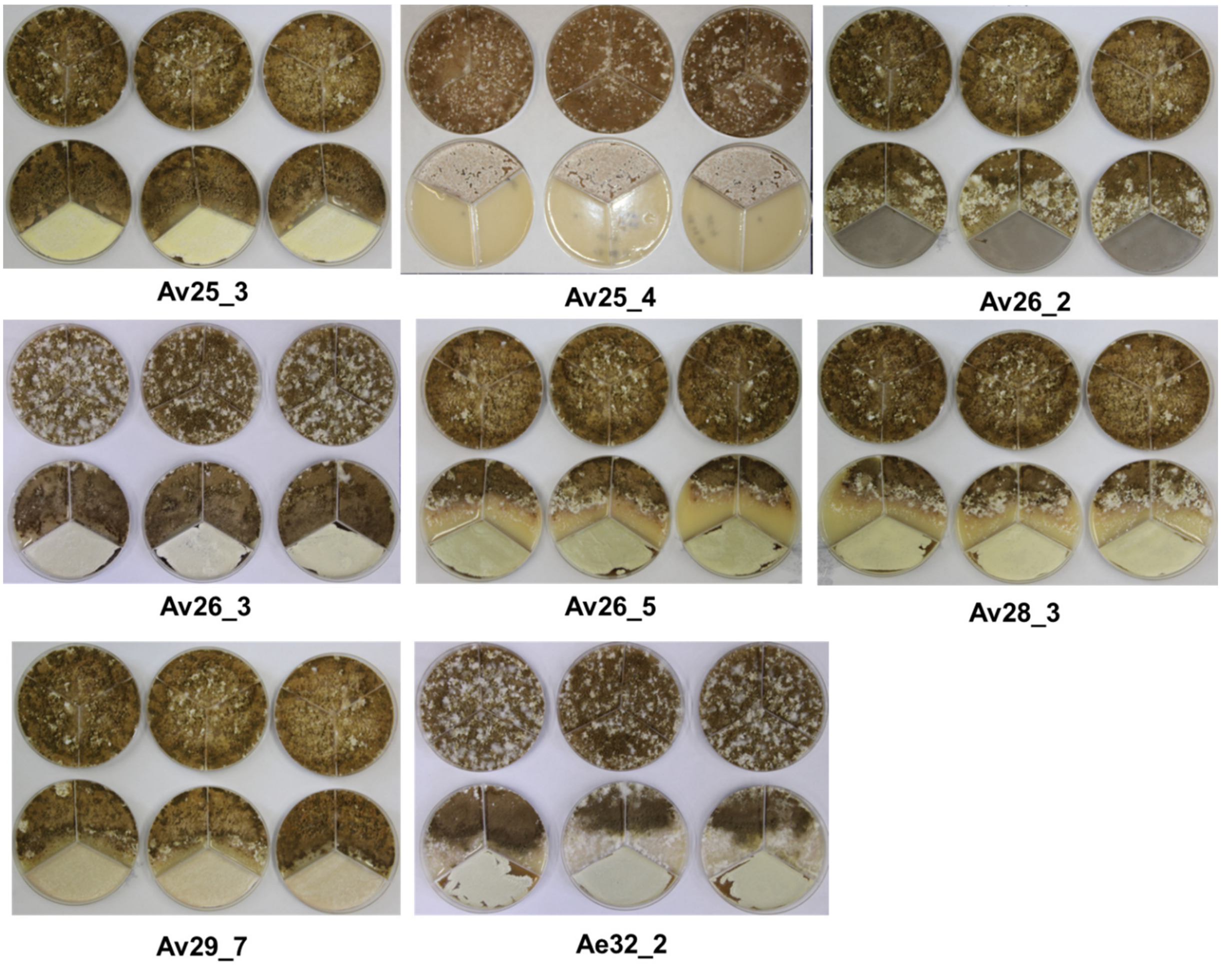

Figure S1. Screening *Streptomyces* symbionts from leaf-cutting ants for the production of volatile antifungal compounds that inhibit the growth of *E. weberi* using the three compartment petri dishes to only allow growth inhibition by volatile compounds. Each picture exhibits the bioassay for one *Streptomyces* symbiont after 10 day of incubation. Three replicates of *E. weberi* control plates (top row) and *E. weberi* co-culture plates (bottom row) are shown in each picture (acronym of name below picture). The tested strains were: *Streptomyces sp. Av25_3, Streptomyces sp. Av25_4, Streptomyces sp. Av26_2, Streptomyces sp. Av26_3, Streptomyces sp. Av26_5, Streptomyces sp. Av29_7, Streptomyces sp. Av28_2, Streptomyces sp. Av28_3,* and *Streptomyces sp. Ae32_2.*

Table S1. *Streptomyces* symbionts used to screen for volatiles with antifungal activity against *E. weberi.*

**Strains**	**Accession number**	**Isolated from**	**Growth inhibition of *E. weberi* mediated by volatile compounds**
Streptomyces sp. Av25_3	FJ490533	*A. volcanus*	+
Streptomyces sp. Av25_4	FJ490534	*A. volcanus*	+++
Streptomyces sp. Av26_2	FJ490537	*A. volcanus*	+
Streptomyces sp. Av26_3	HM538453	*A. volcanus*	No
Streptomyces sp. Av26_5	FJ490538	*A. volcanus*	++
Streptomyces sp. Av28_3	FJ490540	*A. volcanus*	++
Streptomyces sp. Av29_7	FJ490541	*A. volcanus*	+
Streptomyces sp. Ae32_2	FJ490544	A. echinatior	+

+++ very strong inhibition

++ inhibition

+ weak inhibition

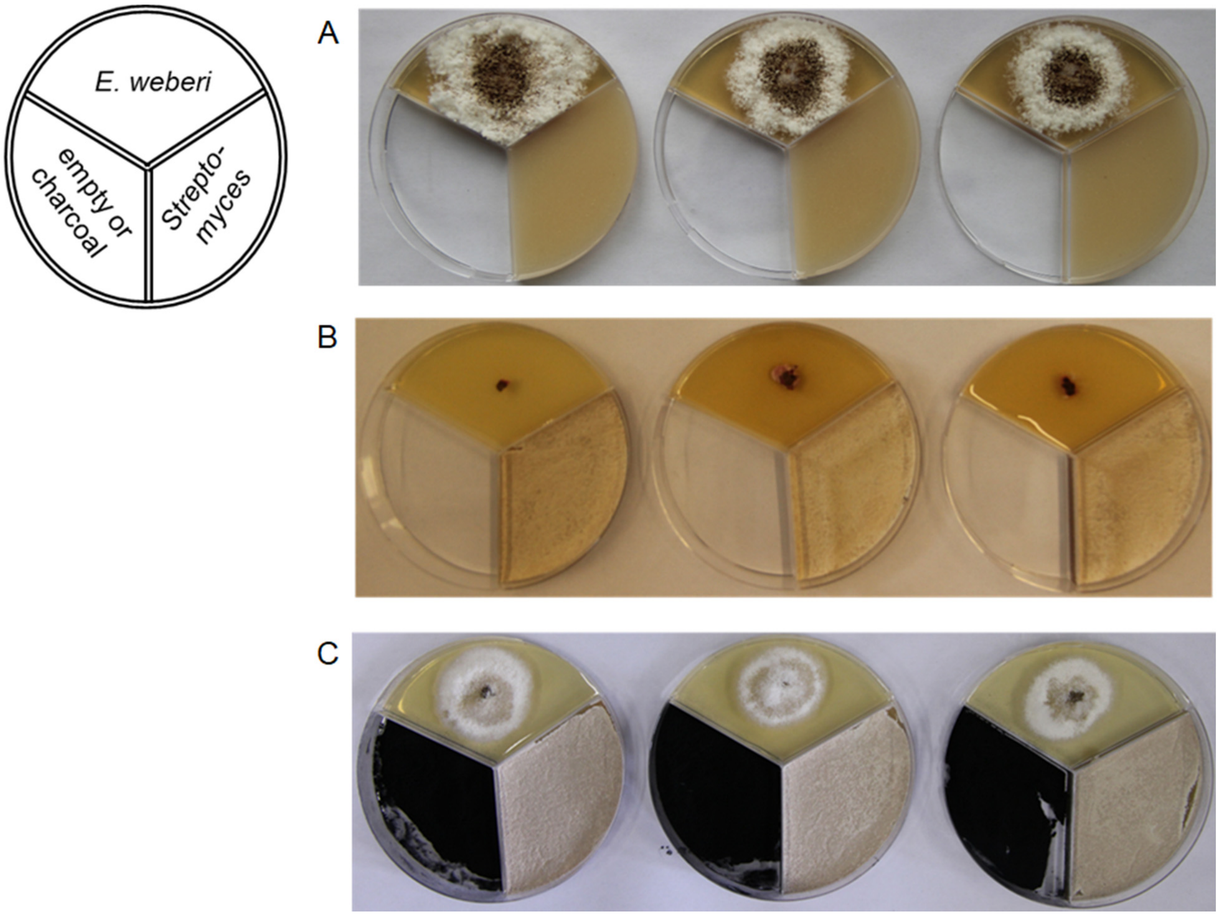

Figure S2. Three compartment bioassay to identify volatile compounds inhibiting the growth of *E. weberi*. A) agar plates with 4 d grown *E. weberi* in the second compartment, SFM medium in first compartment and empty third compartment. B) plates with 4d grown *E. weberi* in second compartment, 7 d grown *Streptomyces* sp. Av25_4 in the first compartment and empty third compartment. C) Agar plates with 4 d grown *E. weberi* in second compartment, 7 d grown *Streptomyces* sp. Av25_4 in first compartment and 2 g charcoal in third compartment. The presence of charcoal led to a reduced growth inhibition compared to the growth of the *E. weberi*/*Streptomyces* sp. Av25_4 co-culture.

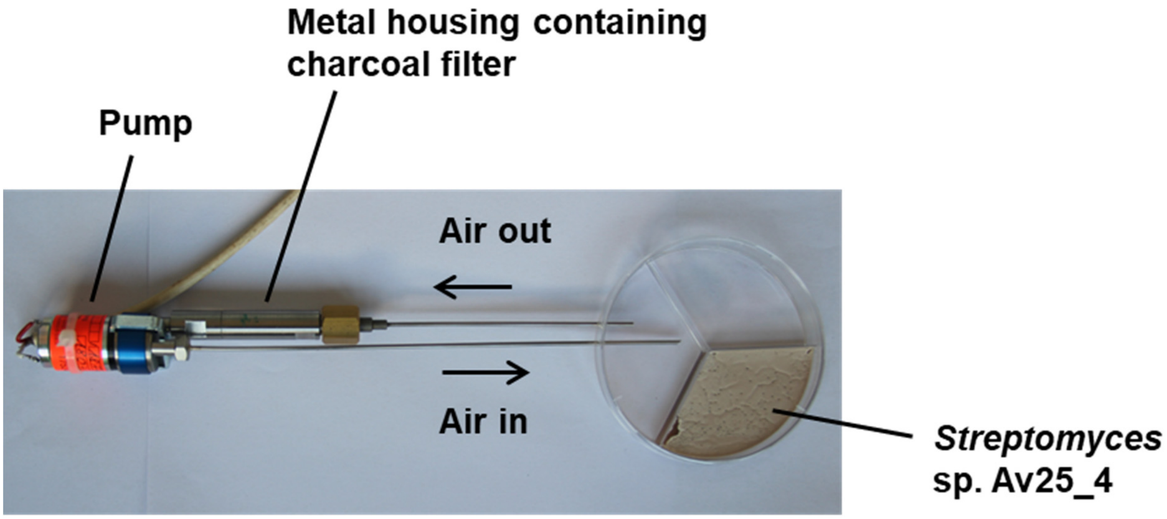

Figure S3. Closed loop stripping. Collection of volatiles produced by *Streptomyces* sp. Av25_4 grown on SFM agar by using closed loop stripping [1].

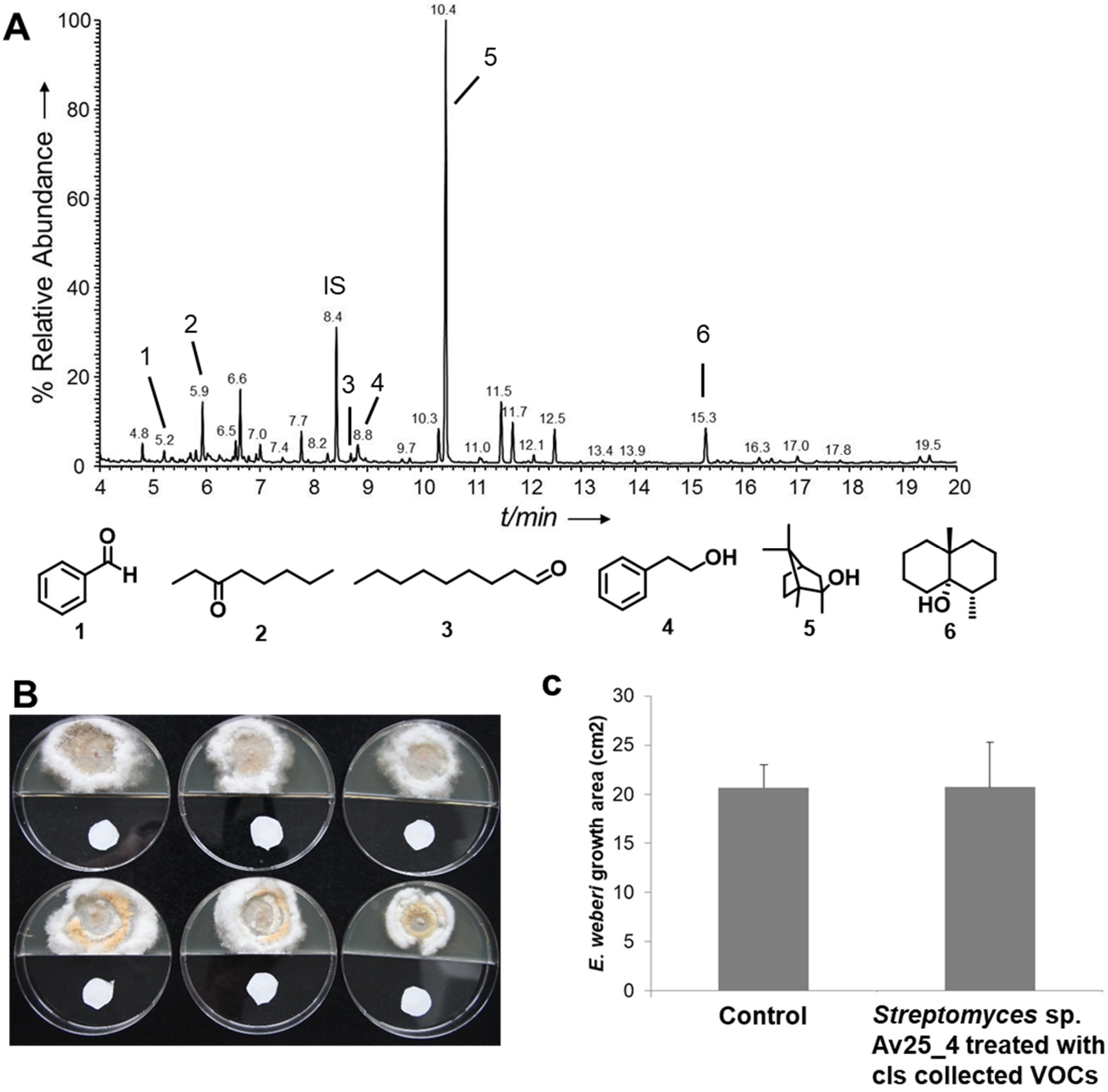

Figure S4. A) Organic volatile compounds from *Streptomyces* sp. Av25_4. GC-MS analysis of collected volatiles produced by *Streptomyces* sp. B) Av25_4 grown on SFM agar. C) Total ion chromatogram of the volatiles produced by *Streptomyces* sp. Av25_4. Structures of identified volatiles produced by *Streptomyces* sp. Av25_4 are given below the chromatogram (IS internal standard – bromodecane).

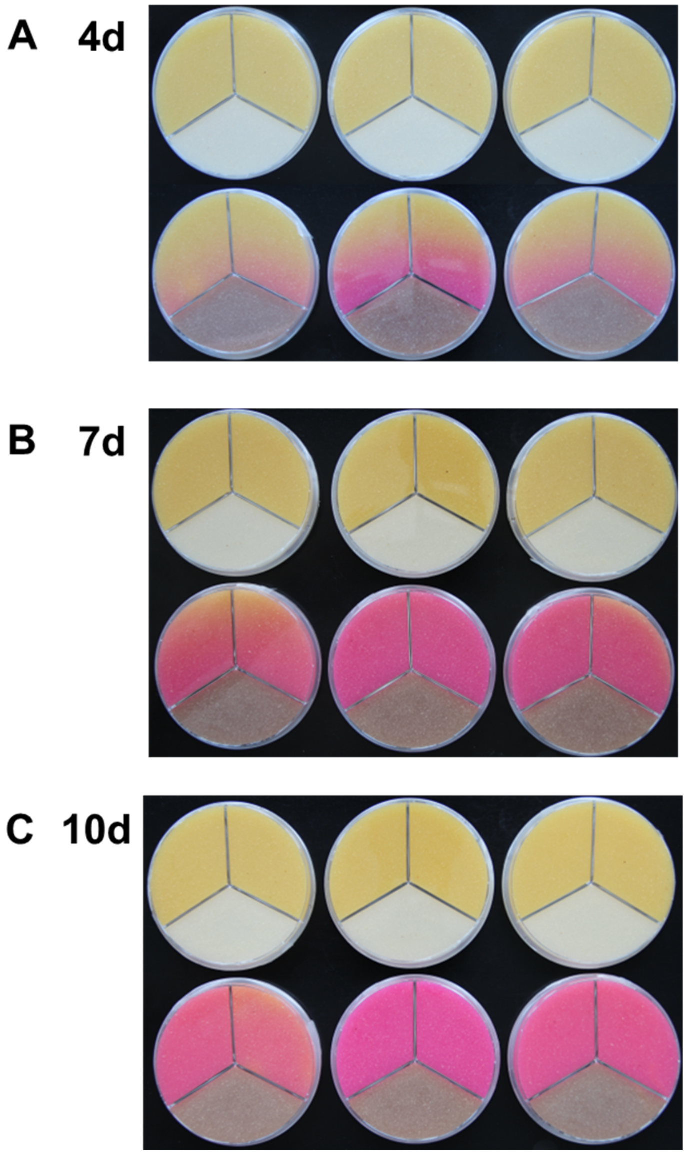

Figure S5. Monitoring pH change in Petri dishes. pH change revealed by the color change of the pH indicator phenol red (pH > 7.3 purple,0.002 %) in the second and third compartment filled with SFM medium [2]. Ammonia produced by *Streptomyces* sp. Av25_4 grown on first compartment induced the pH change in compartment 2 and 3. Top row: control plates with SFM agar in the first compartment and 0.002 % phenol red indicator in second and third compartment. Bottom row: plates with *Streptomyces* sp. Av25_4 growing in the first compartment and 0.002 % phenol red indicator in second and

third compartment. Pictures were taken at day 4, 7 and 10. All samples and controls were performed in triplicate.

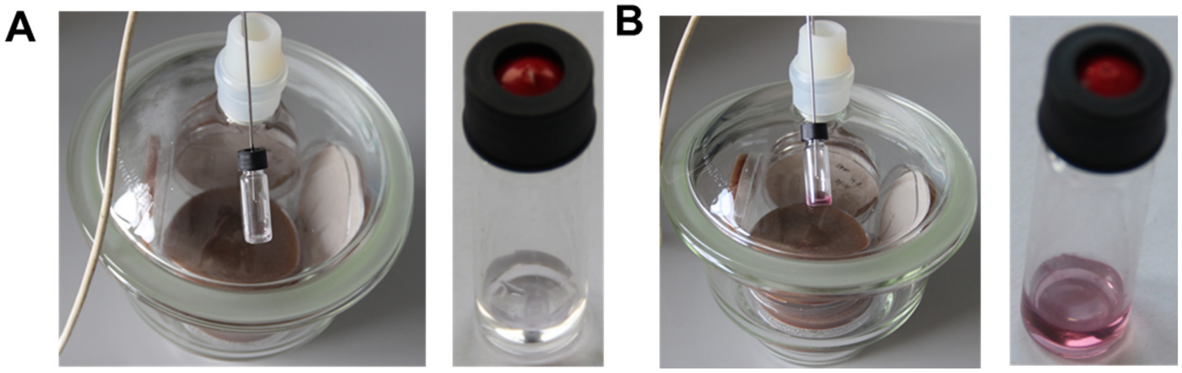

Figure S6. Detection of ammonia produced by *Streptomyces* sp. 25_4 using a closed loop stripping pump transferring the volatiles into the vial with the OPA derivatization reagent. A) Colorless derivatization solution before exposure to the volatiles in the headspace of the desiccator filled with *Streptomyces* sp. 25_4 agar plates. B) Reddish color after exposure of the OPA derivatization reagent to the headspace volatiles indicating the formation of ammonia.

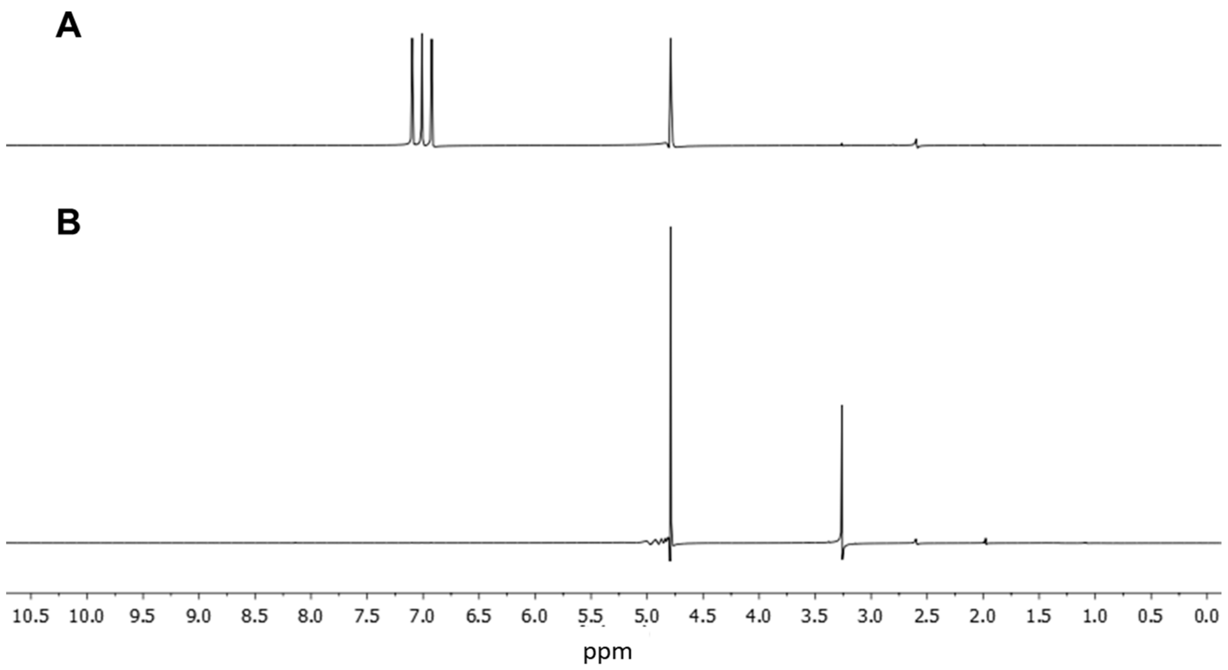

Figure S7. Identification of ammonia as the pH changing principle using ^{1}H-NMR spectroscopy. ^{1}H-NMR spectra (600 MHz) of A) *Streptomyces* sp. Av25_4 produced volatiles exposed 250 mM HCl solution, B) SFM agar plates produced volatiles exposed 250 mM HCl solution as control [4].

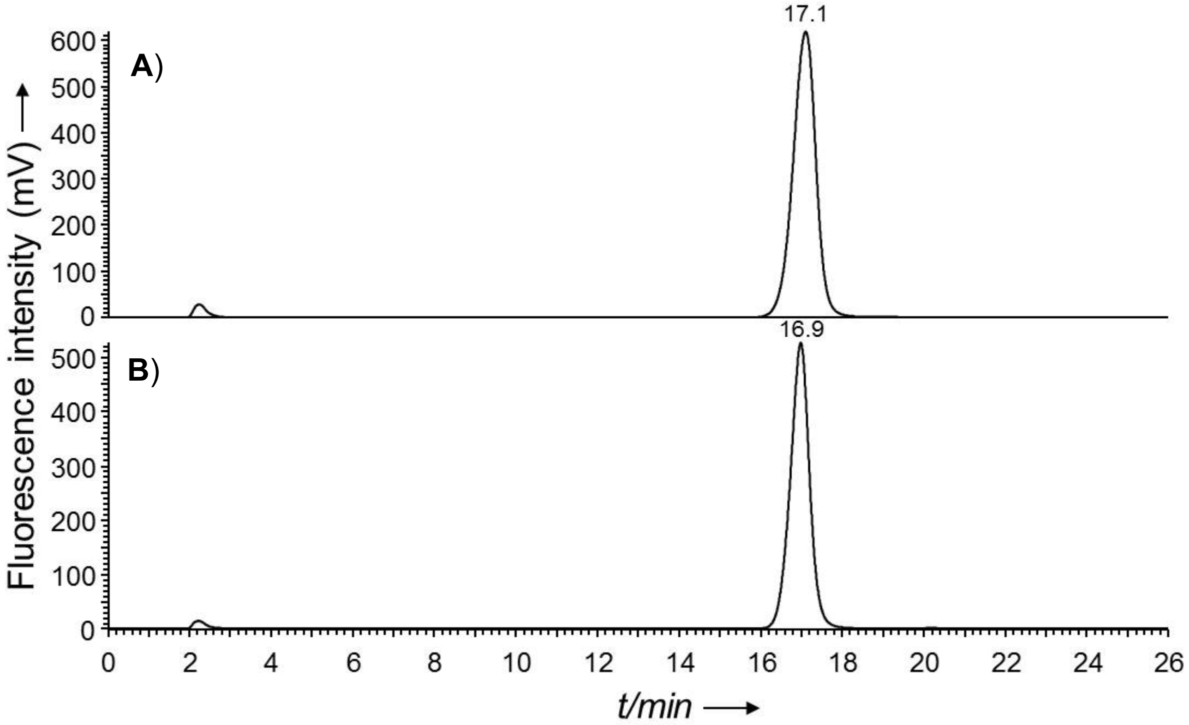

Figure S8. Identification of ammonia as pH changing principle using *ortho*–phthal-aldehyde derivatization [3]. Fluorescence signal measured for the identification of ammonia at excitation and emission wavelength of 360 nm and 420 nm for A) 7d grown *Streptomyces* sp. 25_4 produced headspace volatiles B) 5 mM ammonia standard.

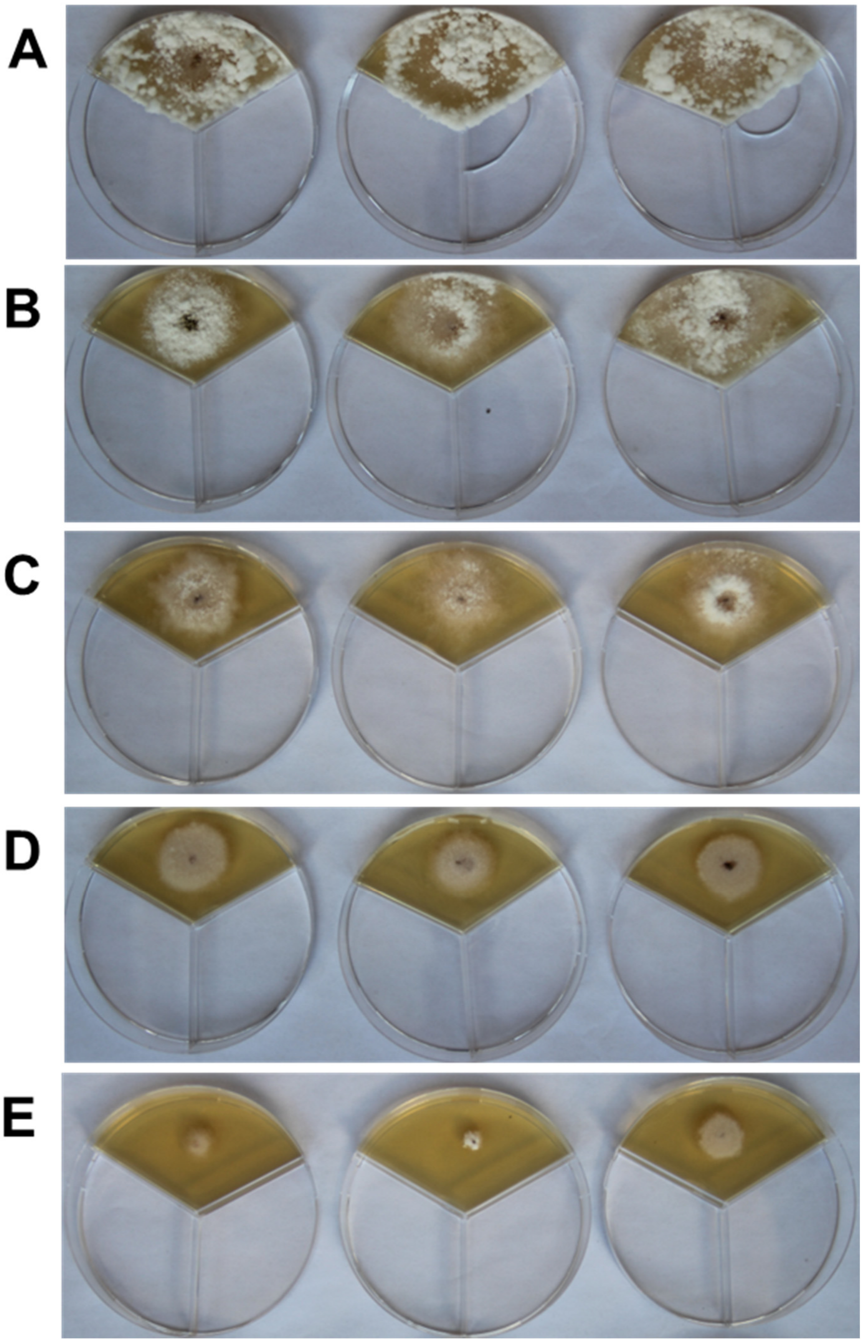

Figure S9. Three compartment Petri dish bioassay evaluating the effect of ammonia on *E. weberi* growth: The first compartment contains 4 d grown *E. weberi*, the second compartment contains 5 ml of sterile water or an ammonia solution in water. The third compartment was left empty. A) sterile water, B) 5 mM ammonia solution, C) 7 mM ammonia solution, D) 9 mM ammonia solution, and E) 11 mM ammonia solution.

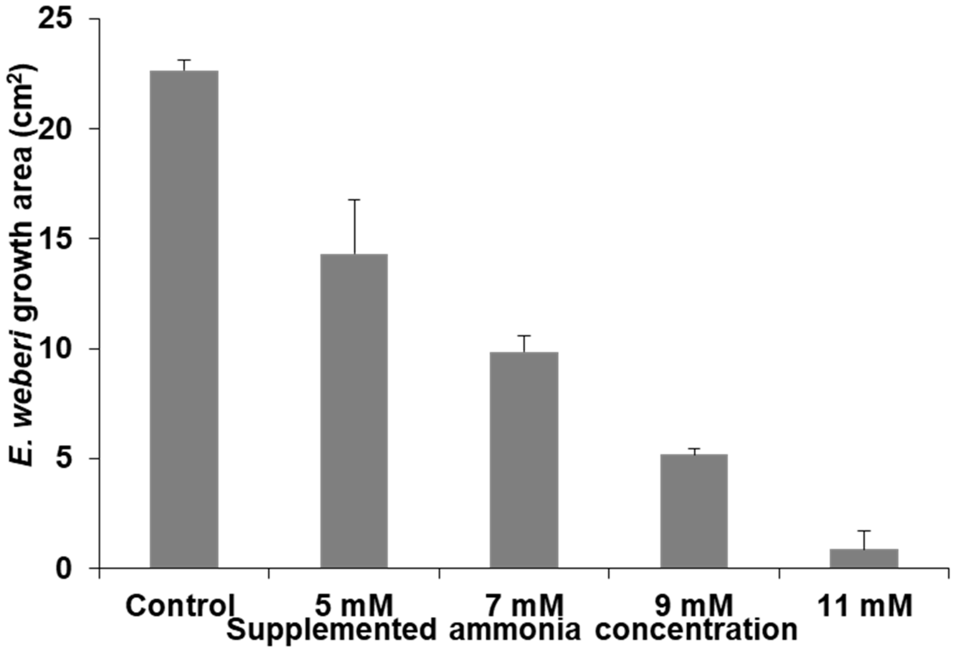

Figure S10. Effect of ammonia on *E. weberi* growth. Quantification of *E. weberi* mycelium growth by analysis of the growth area using image J (3 replicates).

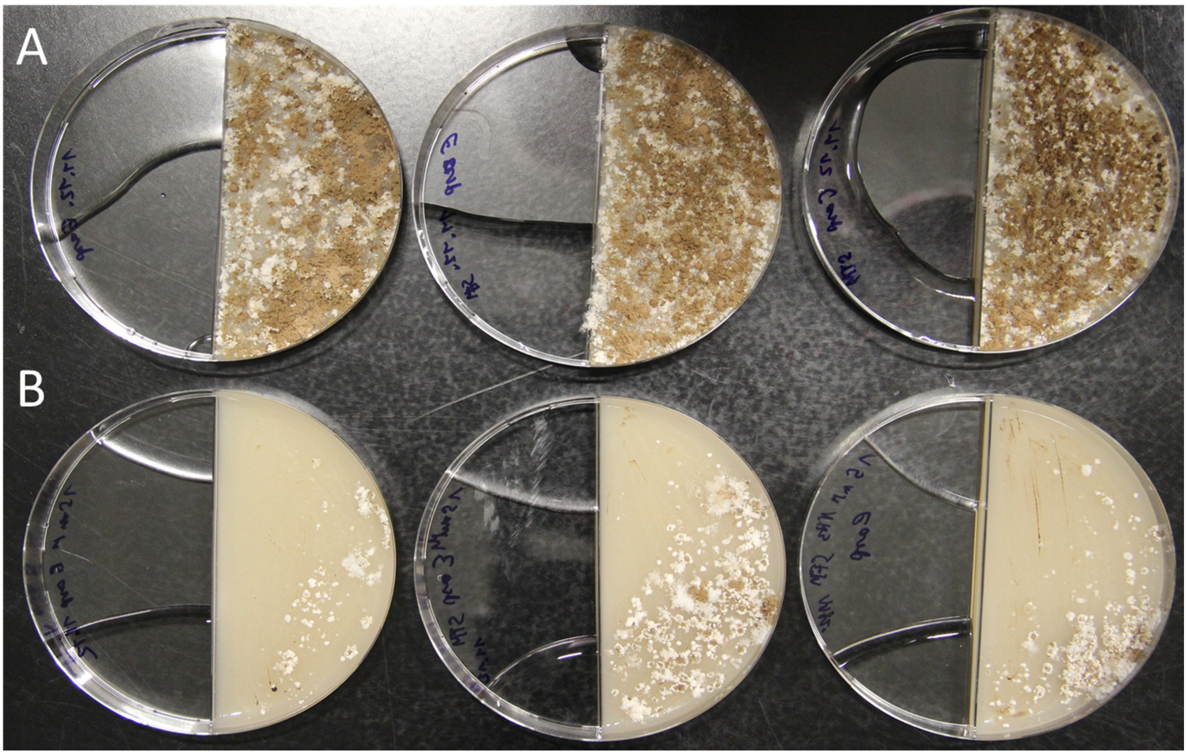

Figure S11. Growth of *E. aspergilloides* in presence of ammonia on SFM agar plates. B) 15 mM ammonia (5 ml) was added into the second compartment on the day of inoculation and A) *E. aspergilloides* without treatment (control). *E. aspergilloides* was grown for 10 d.

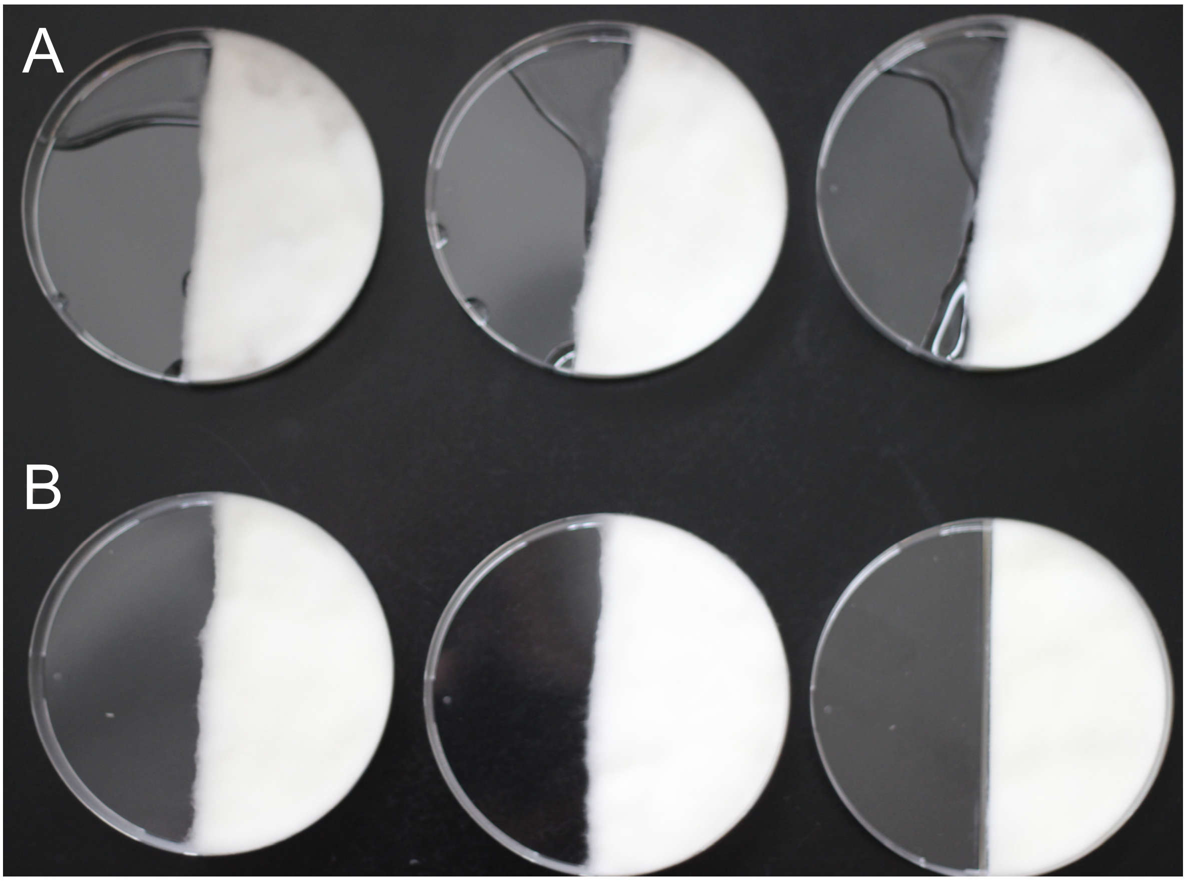

Figure S12. Growth of *F. equiseti* in presence of ammonia on SFM agar plates. A) 10 mM ammonia (5 ml) was added into the second compartment on the day of inoculation and B) *F. equiseti* without treatment (control). *F. equiseti* was grown for 7 d.

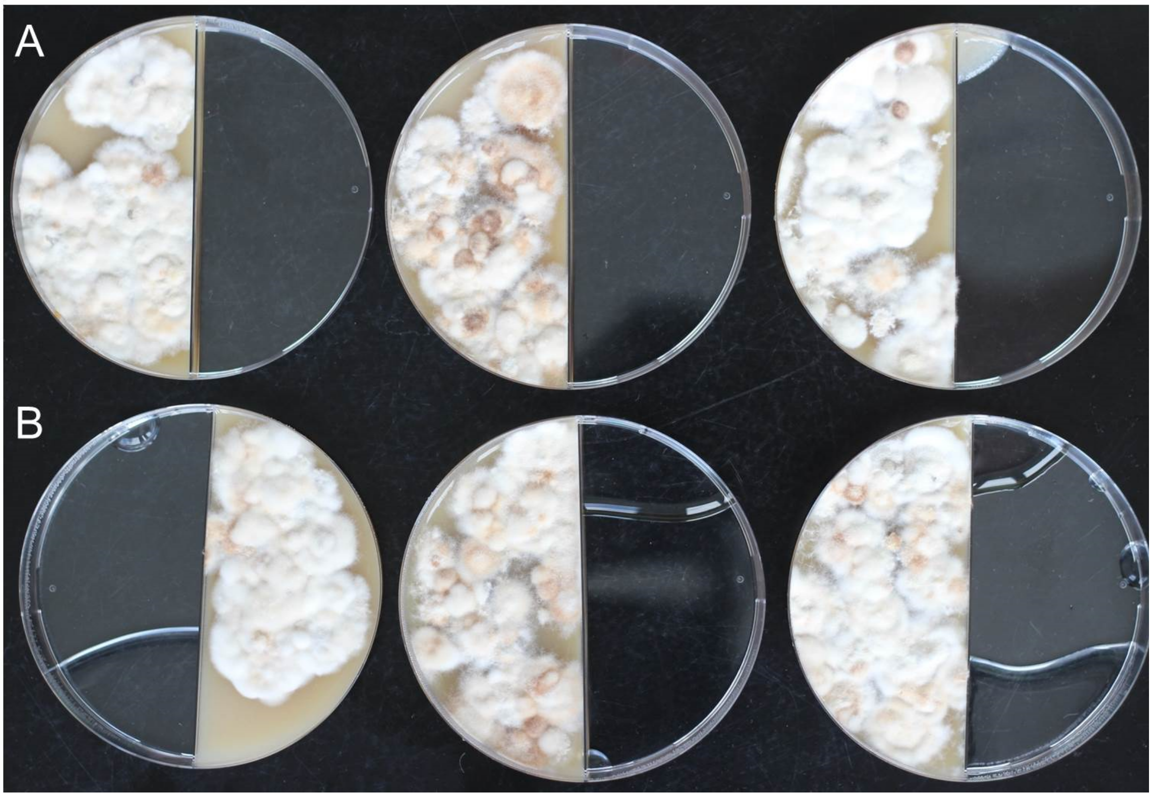

Figure S13. Influence of ammonia on the growth of *L. gongylophorus.* A) *L. gongylophorus* grown on SFM agar plates in one compartment in the second compartment 10 mM ammonia (5 ml) was added on the day of inoculation and B) *L. gongylophorus* without addition of ammonia (control), growth for 3 weeks.

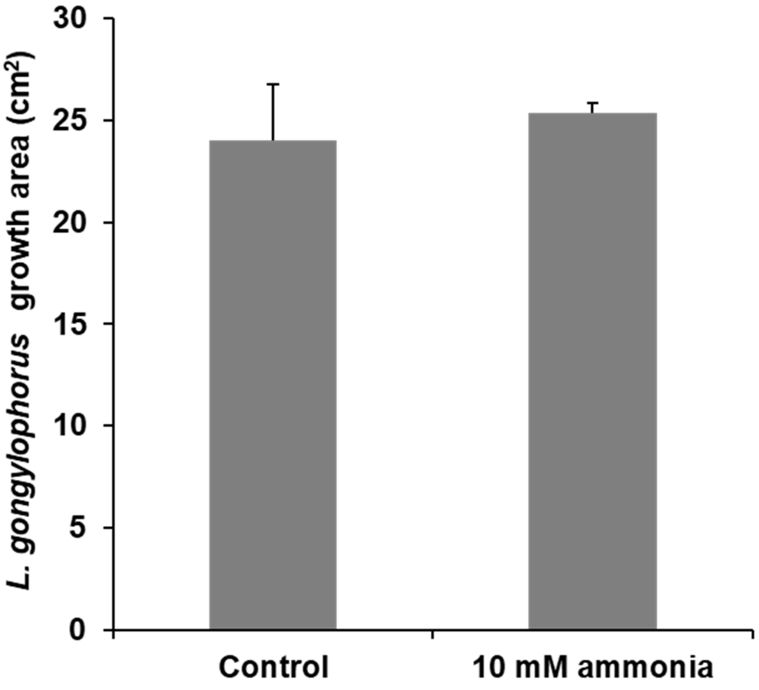

Figure S14. Effect of ammonia on *L. gongylophorus* growth. Quantification of *L. gongylophorus* growth by analysis of the growth area using image J (3 replicates).

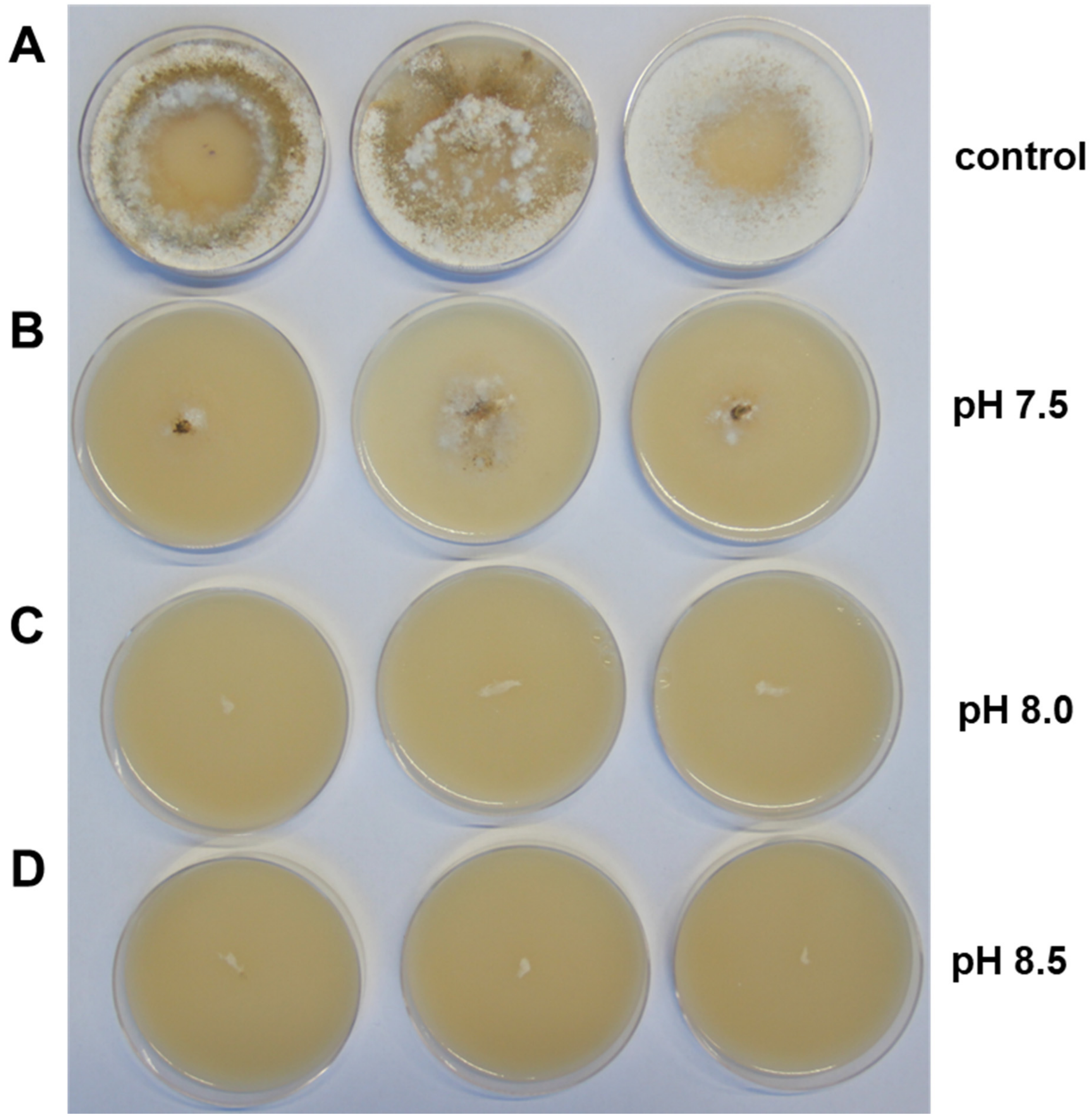

Figure S15. Growth of *E. weberi* on SFM agar plates at different pH adjusted with sodium hydroxide. A) Untreated SFM agar plates (pH 6.2). B) SFM agar plates adjusted to pH 7.5. C) SFM agar plates adjusted to pH 8.0. D) SFM agar plates adjusted to pH 8.5.

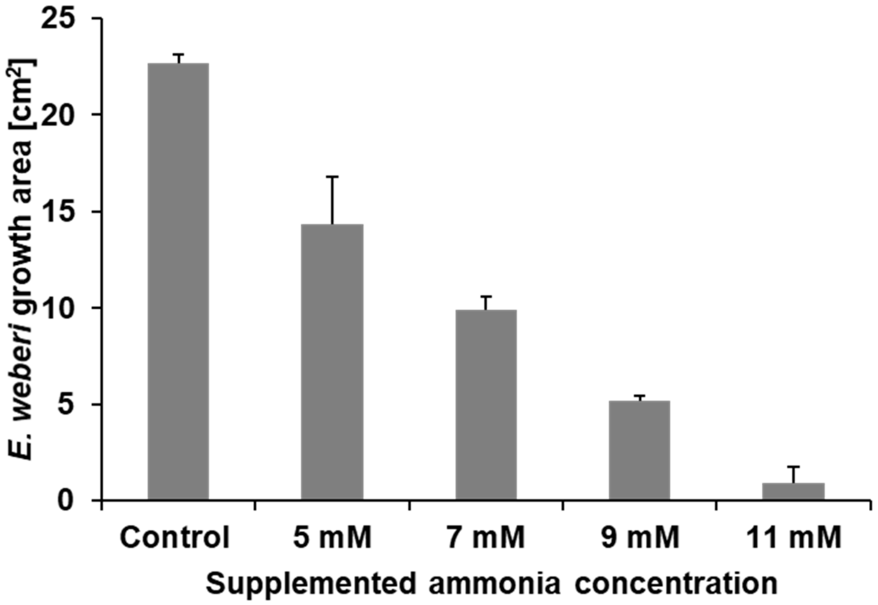

Figure S16. Effect of basic pH on *E. weberi* growth. Quantification of *E. weberi* growth by analysis of the growth area using image J (3 replicates).

Table S2. pH of the waste of different *Acromyrmex* (Hymenoptera: Formicidae: Myrmicinae) and *Atta* (Hymenoptera: Formicidae: Myrmicinae) leaf-cutting ant nests.

**Leaf-cutting ant colony**	**Origin/lab**	**pH**
Acromyrmex ambiguus (Emery, 1888) colony L	Uruguay, Flavio Roces	8.4
A. ambiguus colony T	Uruguay, Flavio Roces	5.2
A. ambiguus colony W	Uruguay, Flavio Roces	5.7
Acromyrmex niger (Smith, 1858)	Brasil, Rainer Wirth	7.7
Acromyrmex octospinosus (Reich, 1793)	Colombia, Rainer Wirth	7.1
Atta laevigata (Smith, 1858) colony Al7	Brasil, Flavio Roces	8.6
A. laevigata colony Al9	Brasil, Flavio Roces	6.9
A. laevigata colony Al11	Brasil, Flavio Roces	8.3
Atta vollenweideri (Forel 1893) colony A	Uruguay, Flavio Roces	5.1
A. vollenweideri colony D	Uruguay, Flavio Roces	5.3
A. vollenweideri colony E	Uruguay, Flavio Roces	8.3
A. vollenweideri colony Konstanz	Argentina, Christoph Kleineidam	8.1
Atta sexdens (Linnaeus, 1758) colony AS1	Brasil, Flavio Roces	8.6
A. sexdens colony AS3	Brasil, Flavio Roces	8.0
A. sexdens colony AS6	Brasil, Flavio Roces	6.3
Acromyrmex lundii (Guérin-Méneville, 1838) colony U2	Uruguay, Flavio Roces	5.0
A. lundii colony U3	Uruguay, Flavio Roces	5.4
A. lundii colony U4	Uruguay, Flavio Roces	4.9
A. lundii colony U5	Uruguay, Flavio Roces	7.3

References

[1] Grob, K. 1973 Organic substances in potable water and in its precursor: part I. Methods for their determination by gas-liquid chromatography. *J. Chrom. A* **84**, 255-273.
[2] Avalos, M., Garbeva, P., Raaijmakers, J.M. & van Wezel, G.P. 2019 Production of ammonia as a low-cost and long-distance antibiotic strategy by *Streptomyces* species. *ISME J*, 1-15.
[3] Genfa, Z. & Dasgupta, P.K. 1989 Fluorometric measurement of aqueous ammonium ion in a flow injection system. *Anal. Chem.* **61**, 408-412.
[4] Schmidt, K. & Spiteller, D. 2017 Ammonia released by *Streptomyces aburaviensis* induces droplet formation in *Streptomyces violaceoruber*. *J. Chem. Ecol.* **43**, 806-816.
